# Developing Sidewalk Inventory Data Using Street View Images

**DOI:** 10.3390/s21093300

**Published:** 2021-05-10

**Authors:** Bumjoon Kang, Sangwon Lee, Shengyuan Zou

**Affiliations:** 1 College of Architecture, Myongji University, Yongin-si 17058, Korea; 2 Department of Human Environment and Design and Department of Human Life and Innovative Design, Yonsei University, Seoul 03722, Korea; sangwon.lee@yonsei.ac.kr; 3 Department of Geography, University at Buffalo, The State University of New York, Buffalo, NY 14260, USA; szou2@buffalo.edu

**Keywords:** sidewalks, GIS, smart street, street management, image processing

## Abstract

(1) Background: Public sidewalk GIS data are essential for smart city development. We developed an automated street-level sidewalk detection method with image-processing Google Street View data. (2) Methods: Street view images were processed to produce graph-based segmentations. Image segment regions were manually labeled and a random forest classifier was established. We used multiple aggregation steps to determine street-level sidewalk presence. (3) Results: In total, 2438 GSV street images and 78,255 segmented image regions were examined. The image-level sidewalk classifier had an 87% accuracy rate. The street-level sidewalk classifier performed with nearly 95% accuracy in most streets in the study area. (4) Conclusions: Highly accurate street-level sidewalk GIS data can be successfully developed using street view images.

## 1. Introduction

The concept of the smart city aims to serve an ambitious goal of optimizing urban resources by observing the usage patterns of city-dwellers. Its application touches a variety of urban infrastructures, such as a smart grid allowing decentralized energy distribution, waste management for detecting and separating trash, monitoring of waste and stormwater, and traffic control for automated vehicles [1]. At the core of the idea are the Internet of Things (IoT) sensors that collect a large quantity of user data for the control of Information and Communication Technology (ICT) devices [2]. This implies at least two crucial opportunities for the industry: massive demand for sensing hardware, and the availability of big data on how people use urban amenities. This explains why giant tech companies in consortium with city governments rush to present blueprints for a rosy future to their constituents.

Despite some small successes, the idea of smart city seems largely unsubstantiated [2]. This is entirely attributable to the ability of the sensors to surveil people’s behavior. Three years ago, a well-known smart city enterprise was initiated when a government agency seeking global investment partnered with a tech company to test AI technology on an urban scale [1]. However, the plan was soon confronted with severe backlash from citizens. Their concerns were the risk of being the testbed of a pilot project and exposing public data for products that will eventually be commercialized. The biggest fear was the technical feasibility of tracing every individual’s entire movements. Even though the company had repeatedly confirmed that the data would be handled as de-identified bulk, the effort was not enough to dispel the deep suspicions rooted by recent data breach cases. The issues raised concerning privacy and ethics seem more than justifiable as sensors and data may fall into the hands of any future regime or company with ill intent.

Upon realizing the public’s negative image of complete smartification by a single entity, this study focuses on sensing the legacy city infrastructure, in particular, the sidewalk. The sidewalk is the primary means of travel and activity of pedestrians, and its safety and comfort are key to the livability of an area [3]. Accordingly, many studies have recognized the sidewalk’s importance in smart city development. They can be categorized into several topics: traffic and pedestrian flow, physical elements of the built environment, and user’s perception of the environment [4]. Recently, studies on the sidewalk have attempted to develop the sidewalk’s new smart applications. For example, one study developed an automatic pedestrian flow detection method to propose a smart management strategy of the sidewalk space [5]. Smart applications of the sidewalk include smart control of the lighting [6], smart street furniture [7], and pavement maintenance [8]. Smart sidewalk systems are expanding and integrating the sidewalks with a smart crossing that can adjust signal intervals for older adults, dynamic curbs that allow driverless vehicles to drop and pick-up passengers, and smart blocks that can change into pavements for either pedestrians or vehicles [1]. Recently, e-scooter companies have developed sidewalk riding detection technologies to protect pedestrians from e-scooter users [9]. Sidewalk detection has many potential applications in smart city development.

To use sidewalks for various smart city applications and developments, researchers need publicly available sidewalk GIS data showing the sidewalk locations, dimensions, coverage, physical conditions, or nearby land use contexts. However, public GIS data for sidewalks are not available in many cities. For example, a study reviewed sidewalk data in a major metropolitan area (King County, Washington State) in the U.S. and found that 12 out of 39 jurisdictions within the area did not have sidewalk GIS data open to the public [10]. When jurisdictions have sidewalk data, their GIS formats were not standardized because each jurisdiction developed its data for its own purposes. Therefore, sidewalk GIS data should be processed for standardization across jurisdictions to develop a larger-scale dataset [11]. As a basic preparation, it is necessary to have standardized, global, and public sidewalk GIS data across administrative borders.

Standardized street view images may be useful if it is possible to extract sidewalk information from street view images provided by private or crowdsourced services such as Google Street View (GSV), Apple Look Around, the Mapillary project, and KartaView. Many studies have used GSV images to extract street-side information for street facility assessment or street behavior detection [12,13,14,15,16]. Because street view images are available in many countries and have similar image formats, they can be used to produce sidewalk data. 

The current study aims to develop an automated method to develop sidewalk GIS data using computer vision and machine learning with extracting sidewalk information from Google Street View (GSV) images.

## 2. Materials and Methods

**Figure 1:**
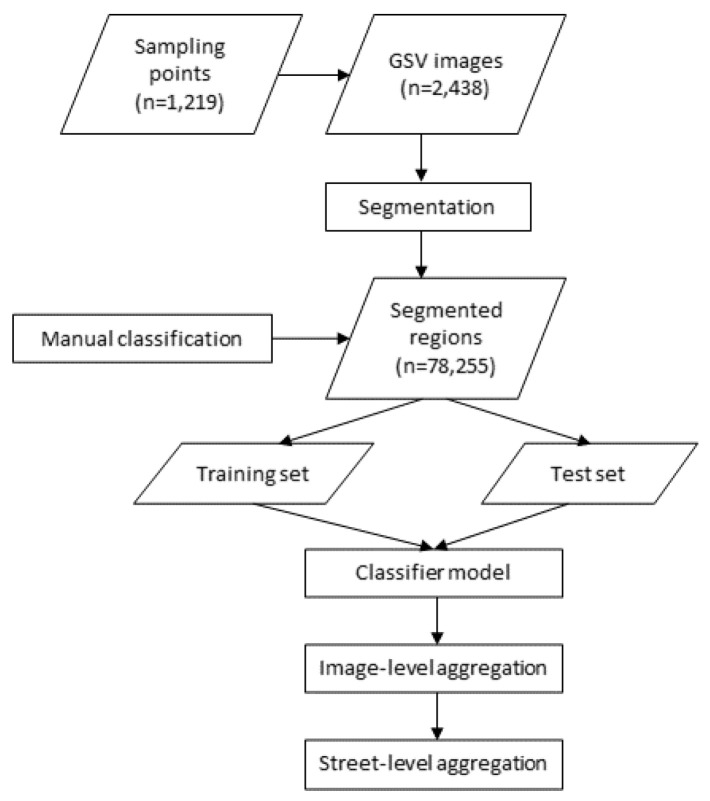
Street-level sidewalk classification process.

The overall process is illustrated in Figure 1. Detailed methods are explained in the following sections.

### 2.1. Sample Points

Street view services provide street images given a point on the street. We developed our study sample of streets, using the following procedure. First, we selected street segments from the study area, defined as Erie and Niagara Counties, NY, covering urban and rural areas in the Buffalo–Niagara Falls metropolitan region. In the study area, public street network segments were used from the street network dataset built from the 2013 TIGER Census street line files, excluding ones that were non-traversable by pedestrians (e.g., highways or onramps). This step collected all street segments possibly having sidewalks. Second, street segment polylines were split at every vertex and at a 30-ft interval distance. Because most streets have curb cuts or partial sidewalks, we needed to determine the sidewalk presence at multiple locations on a street segment. After initial screening GSV images along continuous streets, the 30-ft interval was chosen to avoid image overlaps between adjacent GSV images. For each split segment, we characterized its geometric properties including the azimuth, calculated by the angle of the split street segment’s orientation. Third, we generated each split segment’s center point where street images will be collected, yielding a total of 1,393,294 (center) points. Finally, from the generated points, we randomly sampled 2000 points as our study sample set. Each point has its latitude and longitude information and the azimuth of the street that the point generated from. Sampled points were used as locations where GSV images were collected via Street View Static application programming interface (API).

### 2.2. Eye-Level Street Images

Out of the 2000 sample points, GSV images were available at 1632 points. In addition, we removed invalid points where we did not need to determine the sidewalk’s presence. We decided to exclude 413 street points located within 50 ft of street intersections because they legitimately have no sidewalks. At each point, the left and the right side of eye-level street images were obtained using GSV Static API [13]. Using the street azimuth information, we were able to specify the left and the right side of a street point (subtracting or adding 90° to the street’s azimuth). The field of view was set to 120° and image size was set to 640 pixels by 640 pixels. Finally, we have 1219 points, yielding the GSV study sample consisting of 2438 GSV images in the left and right side of their streets. The final sample served to develop an object-based classification algorithm to detect sidewalks. GSV images were obtained within Python 3.7 and street segment azimuth calculation was conducted with PostgreSQL and PostGIS 2.0.

### 2.3. Image Pre-Processing

First, GSV images were standardized by being cropped to their lower halves, in order to reduce image variation in sidewalk detection and to focus on sidewalk areas. Second, for each GSV image, pixels were grouped into discrete regions using a graph-based image segmentation method [17]. A region in a GSV image was treated as a unit to label whether or not it was a sidewalk. The graph-based segmentation method considers a pixel as a node connected through edges to its neighboring nodes (pixels) in a graph and groups pixels into regions based on pixel similarity. To have a better performance in segmentation, we tested the *K*-Nearest-Neighbor method with different parameter sets with a random sub-sample (sample size = 100; ~4% of the GSV study sample). When the Gaussian smoothing filter parameter σ = 0.5 and the size of nearest neighbors *K* = 10, the segmented results have fewer over-segmented regions and less noise in the region boundaries. Third, segments with <1000 pixels were considered as noise and were excluded from the analysis. Finally, we applied a dilation operation for removing noise exiting in the boundary between regions.

### 2.4. Feature Extraction

For each image region, we extracted features from the following four perspectives: its relative location in the image, geometric features, color, and the number of lanes contained within the region, explained below.

- Location: Horizontal (x) and vertical (y) distance in pixels from the bottom left corner of the image to the segment’s centroid were calculated.
- Geometric features: A segment’s size (number of pixels in the region) and perimeter (number of pixels in the boundary) in pixels were measured. The equivalent diameter, defined as the diameter of a circle with the same area of the region, was calculated. We calculated a segment’s orientation from the major axis orientation to fit the segment’s boundary. The shape irregularity was measured as the number of vertexes of the segment’s minimum convex hull. In general, sidewalk segments tend to have more regular shapes (close to rectangles or parallelograms) than other types of segments (e.g., trees).
- Color: A segment’s color was characterized using a standard color library (SCL) for sidewalks. We randomly sampled 10 true sidewalk segments from our study sample dataset and created their standard RGB (red, green, and blue) and HSV (hue, saturation, value) histograms. For each region, histograms for each six color bands were created and measured for their cumulative distances from the SCL histograms, yielding six color features.
- Number of lanes: for each GSV image, the number of lanes were estimated with the following steps. First, images were sharpened using three morphological elements for three major directions (45°, 90°, and 135°), to create easily recognizable contrasts around potential lanes.

Second, we used the *Hough* transformation, an invariant edge detector method, to detect edges of lanes [18]. Third, Hough peaks (i.e., local maximums) were identified as lane vertexes. The parameter of Hough peaks was set as 2, based on our review on sample images. Fourth, the maximum value of the gap between two connected peaks was set as 5 and the minimum length of each lane was set as 80 pixels to remove small noises, based on reviewing and testing with the sample images. Finally, the number of lanes were calculated from the detected lane vertexes.

In total, 14 features were extracted and their values were normalized between 0 and 1, with a linear transformation using min-max scaling.

### 2.5. Labeling Ground Truth

**Figure 2:**
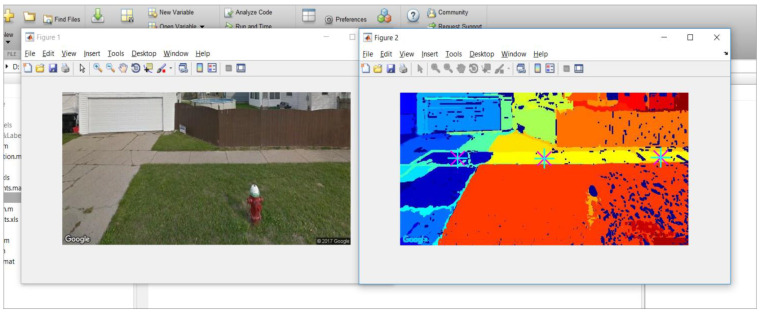
Custom-built software labels sidewalks by visually inspecting the original and segmented images side-by-side. The software asks an analyst to put the pointer where the sidewalk is present and labels the associated segment.

We had two analysts to visually inspect all image regions processed above, to label them as sidewalk or non-sidewalk. A GSV image with a sidewalk region was labeled as a sidewalk GSV image. The manual labeling results served as the ground truth dataset to train and test the classification models explained below. A custom-made interactive tool, using MATLAB, was developed (Figure 2). The tool displays the original GSV image and its segmented image side-by-side. Analysts were trained to point to all sidewalk regions in the given GSV image. Then, the tool labeled the indicated segments as sidewalks and the rest of the segments as non-sidewalks. Figure 2 shows a screen capture of the tool with a GSV example. GSV images with a region labeled as sidewalk were considered as sidewalk GSV images.

### 2.6. Training, Testing, and Classifier Model

Once segments were labeled and sidewalk GSV images were determined, we established a training and a test set with a two-step process. First, we split all GSV images into a training image group (two-thirds of the entire sample) and a test image group (one-third, the rest of the entire sample). The groups were stratified with respect to the ratio of the number of sidewalk and non-sidewalk GSV images. Second, we developed a segment-level training set, consisting of all segments from the image-level training group and a segment-level test set from the image-level test group.

We established a random forest model, using the segment-level test set, classifying a segment as sidewalk or non-sidewalk. The random forest model, based on a machine-learning algorithm, has been widely applied in various areas including image classification for its high accuracy, robustness, and low possibility of overfitting [18,19]. Previous studies used random forest models to classify or label image segments in GSV images [20,21]. The training set had imbalanced classes (more non-sidewalk than sidewalk segments) and may yield a low prediction accuracy of the minority class. To address the issue, we down-sampled the majority class (non-sidewalk) to the same sample size of the minority class (sidewalk class) and adjusted voting thresholds [22]. We applied varying levels of the voting threshold and selected the final model that produced the lowest within-class errors in both classes.

### 2.7. Aggregation at the Image and Street Levels

Using the selected model, each segment’s probability of being sidewalk was estimated. If a GSV image had a segment with a sidewalk probability equal or greater than *R*, the sidewalk image determination threshold, the GSV was considered to have sidewalk. Otherwise, it was considered as a non-sidewalk GSV image. Low *R* values may produce false positive errors and high *R* values may produce false negative errors. We conducted a sensitivity analysis to find an adequate *R* value. We applied *R* values between 0.1 and 0.95 at a 0.05-interval and measured rates of false positive, false negative, and overall errors, using the training set. The *R* value with the lowest error rates was selected and then applied to the image-level sidewalk classification. We obtained the overall lowest error rate.

**Figure 3:**
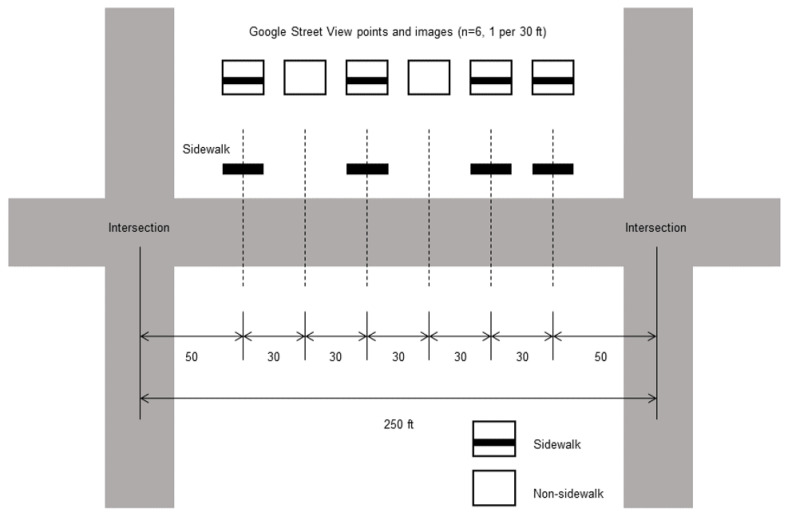
Street-level sidewalk classification illustration.

Our aim was to determine sidewalk presence or absence of a side of a street. Potential errors at the image level may be reduced and are aggregated into a larger group, at the street level. For example, a 250-foot street has 6 GSV image points at 30-foot intervals, for one side, excluding two end portions with a 50-foot distance (Figure 3). The image-level classifier developed above determines the sidewalk presence of each of 6 images with an error rate. A street’s side is determined as having sidewalk when half or more of the GSV images are classified as sidewalk images. In the example, 4 out of 6 images are classified as sidewalks, which determines the street as a sidewalk street on the site studied.

We assumed that the image-level classifier has an error rate *E*, which is the lowest error rate of the classifier above. With varying lengths of streets, we conducted 1000 simulations of street-level sidewalk classifications to assess how different levels of *E* affect street-level classifications of different street lengths. We examined the distribution of streets in New York State, using topologically-corrected and simplified OSMnx street network data [23]. 

We used R version 3.5.1 (R Foundation for Statistical Computing, Vienna, Austria) and R randomForest package for establishing random forest models.

## 3. Results

**Figure 4:**
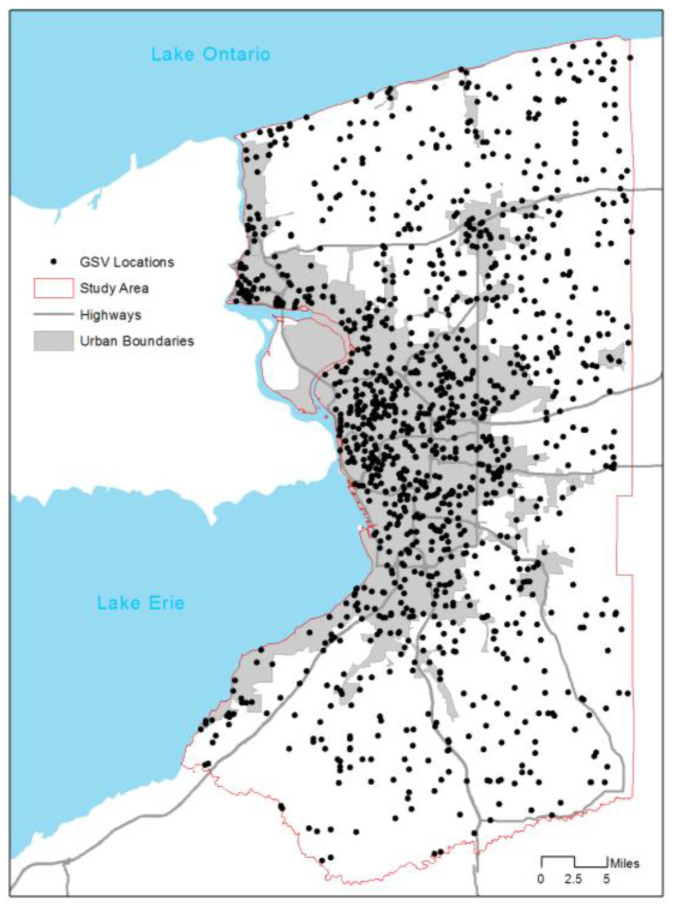
Sidewalk street image points and the study area.

**Figure 5:**
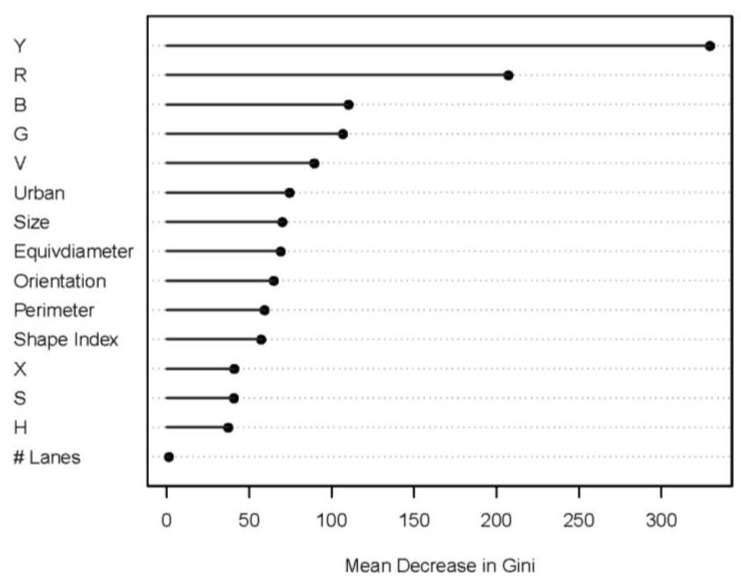
Importance of variables in the random forest model.

In total, the study area included 79,875 street segments, yielding 1,393,294 center points of split segments. Figure 4 shows locations of GSV image points and the study area. The image points were located mostly within urban boundaries and a small portion in low-density rural areas. From 1219 points, we collected 2438 GSV images in the left or right side of streets. The image pre-processing applied the graph-based segmentation for the all images, yielding a total of 78,255 segmented image regions. Of the regions, 2002 were manually labeled as sidewalks and 76,253 as non-sidewalk regions. Next, we extracted features of the sidewalk image regions and non-sidewalk image regions. Table 1 shows descriptive statistics of the regions. On average, sidewalk regions located in the upper parts in GSV images (Y mean: 617.95 vs. 331.62) have high values in some RGB and HSV histogram distributions, and tend to have regular shapes, small sizes, large perimeters, and small number of lanes, compared to non-sidewalk regions. We established a random forest classifier model using the features. Figure 5 shows the importance of features (variables) of the segment-level random forest classifier model. As expected, the horizontal location, and R, B, G, and V histogram distributions had the top five important features, meaning the location and the color composition are more important than others in detecting sidewalk image regions. 

Image regions were aggregated at the GSV image level. At the GSV image level, we conducted a sensitivity analysis of the sidewalk error rate with respect to *R*, the sidewalk image threshold. It turns out that the *R* value of 0.9 yields the lowest overall error rate (8%) in the training set and the lowest (13%) in the test set (Table 2). In the test set, 13% of the errors are from 7% of false negative errors and 6% of false positive categories. 

Finally, we aggregated the GSV image-level sidewalk information into the street-level information, to determine the sidewalk presence or absence of a street. Table 3 shows the street-level sidewalk detection accuracy values with respect to the street lengths and the hypothetical image-level sidewalk detection error rates. With an image-level error rate (*E*) of 13% (when *R* is set to 0.9), the street-level sidewalk detection accuracy is over 95% when the aggregated GSV images are 2 or more (or the street length is ≥130 ft). Thus, when a street is 130 feet or longer, which is 91.7% of cases, the sidewalk detection accuracy is always over 95%. This is true even when the image-level error rate increases from 13% to 19%. When a street is 190 feet or longer (meaning the street has 4 or more GSV image points), street-level sidewalk detection is nearly 99% accurate under the image-level error rates of 0.13. In New York State, 13.9% of all streets were shorter than 190 feet. Thus, for most streets (86.1%), this street-level classifier successfully determines if a street has sidewalk (99% accuracy).

## 4. Discussion

The current study used a graph-based image segmentation method to build street-level sidewalk GIS data using GSV images [16]. This approach is relatively simple. It uses a small size of training samples, not requiring high computing power nor highly sophisticated techniques. This is because our approach used multiple aggregations (segmented regions to images and images to streets). At the segment level, accuracy levels may not be high enough. However, when they are aggregated, errors are canceled out by successful classifications. For the purpose of sidewalk inventory development, we just need street-level sidewalk presence information. The current study showed that our method successfully and sufficiently presents excellent accuracy rates (95–99%). 

The study’s approach has several strengths. First, the method can be applied to any areas where GSV services are available. The study area covers a wide range of urban and rural regions having sufficient spatial variability. Second, our approach may be useful even in areas where GSV services are not available, yet where some other similar services are available. GSV data formats are similar across providers. Given a location, a user can retrieve street images with parameters, which can be translated across service providers. For example, a study used GSV data to detect street-side greenery [21]. Another study used Baidu Street View data [24]. They both have common approaches. Third, this method will substantially save monetary and time cost in building sidewalk GIS data. In our study, it took about 24 person-hours to manually label sidewalks in about 2500 GSV image samples. It would take 560 person-hours if we manually labeled the entire 1.4 M sidewalk image points from the study area of 2400 mi^2^ (≈6200 km^2^). Manual labeling usually requires additional time and costs for cross-validation and data management, meaning that a larger dataset means even larger labor and costs. The current automated method reduces considerable time and cost of data development. Furthermore, the method is reproducible. Thus, it may be used for continuous sidewalk data management.

The current study presents a relatively simple method to develop sidewalk inventory data using publicly available or commercial street view data. We tested the reliability of the accuracy value of our approach, using five-fold cross validation. The five sets of validation yielded accuracy values with a 2.88%-point difference between the minimum and maximum accuracies (data not shown), suggesting that our image-level accuracy possibly increases or decreases by that difference. However, such changes in accuracy do not affect street-level outcomes, as shown in Table 3.

Our method used a graph-based image segmentation. We did not consider advanced image-processing methods like Deep Learning because they usually require a large training sample. For the current purpose of sidewalk detection, the graph-based method is sufficient and feasible to produce an excellent agreement rate over 95%.

Our study may have limitations. Like other studies using image recognition, the current study’s performance depends on the quality of source images. We observed from random spot checking that some large shades in street images may lead to mis-classifications. Shades tend to make multiple objects into one object. We reviewed potential methods to restore shades or shadows. However, we determined not to apply the restoration method because we had sufficiently high success rates in our data without using it. Future studies may need to apply shade restoration techniques when shades significantly affect classification performance.

Recent studies use Light Detection and Ranging (LiDAR) sensors to detect street facilities [25,26]. LiDAR sensors collect direct data and may capture precise details of the street environments. Future studies are needed to integrate street view image data with LiDAR-collected information to assess street environments. 

The current study focused on the sidewalk. Comprehensive street assessment may be possible when we have additional information as neighborhood qualities and conditions [27,28]. Objective data methods like our study may contribute to build smart street management and neighborhood planning strategies.

## 5. Conclusions

We developed an automated method to detect sidewalk presence at the street level using Google Street View data. The sidewalk detection at the image level shows an acceptable accuracy rate of 87%. When the sidewalk information is aggregated into the street level, which is practical for smart sidewalk management purposes, the sidewalk detection shows excellent accuracy rates ranging 95–99%. Street images are useful to produce highly accurate sidewalk GIS data. 

## Figures and Tables

**Table 1:** Segmented image region: descriptive statistics before standardization.

Variables		Sidewalk Regions (*n* = 2002)		Non-Sidewalk Regions (*n* = 76,253)	
		Mean	SD		
Location	X	132.14	38.00	Location	X
	Y	617.95	180.78		Y
Color	R	0.41	0.15	Color	R
	G	0.45	0.17		G
	B	0.38	0.16		B
	H	0.50	0.23		H
	S	0.22	0.13		S
	V	0.38	0.17		V
Geometric	Shape Index	73.67	48.75	Geometric	Shape Index
	Orientation	−0.28	11.11		Orientation
	Size	4442.10	5316.13		Size
	Perimeter	407.01	337.94		Perimeter
	Equivalent diameter	67.69	32.77		Equivalent diameter
Number of lanes		0.02	0.20	Number of lanes	

**Table 2:** Error rates by varying *R* (the sidewalk image determination threshold) in the training and test sets.

	Training Set (1622 Images from 52,471 Segments)			Test Set (813 Images from 25,784 Segments)		
*R*	Error Rate			Error Rate		
	False Negative	False Positive	All	False Negative	False Positive	All
0.10	0.67	0.00	0.67	0.67	0.00	0.67
0.15	0.55	0.00	0.55	0.56	0.00	0.56
0.20	0.49	0.00	0.49	0.48	0.00	0.48
0.25	0.44	0.00	0.44	0.45	0.00	0.45
0.30	0.40	0.00	0.40	0.40	0.00	0.41
0.35	0.36	0.00	0.36	0.38	0.00	0.38
0.40	0.33	0.00	0.33	0.35	0.00	0.35
0.45	0.29	0.00	0.29	0.30	0.00	0.30
0.50	0.26	0.00	0.26	0.28	0.00	0.28
0.55	0.24	0.00	0.24	0.25	0.01	0.25
0.60	0.21	0.00	0.21	0.22	0.01	0.23
0.65	0.18	0.00	0.18	0.19	0.01	0.20
0.70	0.15	0.00	0.15	0.15	0.02	0.17
0.75	0.13	0.01	0.13	0.13	0.03	0.16
0.80	0.10	0.01	0.11	0.11	0.03	0.14
0.85	0.08	0.02	0.10	0.09	0.04	0.13
0.90 *	**0.04**	**0.04**	0.08 *	**0.07**	**0.06**	**0.13**
0.95	0.01	0.07	0.09	0.03	0.11	0.15

* Selected *R* based on the lowest in-class and overall error rates in the training set (shown in bold).

**Table 3:** Street-level accuracy rate given different levels of image-level accuracy 0.79 (*E* = 0.21) and 0.87 (*E* = 0.13).

Number of GSV Images [Count]	Street Length [ft]	Length Distribution in the Study Area	Street-Level Accuracy [%]				
		Accumulated Frequency [%]	Where Image-Level Error Rate (*E*) = 0.13 * (Given *R* = 0.90)	*E* = 0.15	*E* = 0.17	*E* = 0.19	*E* = 0.21
0	<100	6.3	NA	NA	NA	NA	NA
1	≥100, <130	8.3	0.87	0.85	0.83	0.81	0.79
2	≥130, <160	11.0	0.98	0.98	0.97	0.96	0.96
3	≥160, <190	13.9	0.95	0.94	0.92	0.91	0.89
4	≥190, <220	16.7	0.99	0.99	0.98	0.98	0.97
5	≥220, <250	20.4	0.98	0.97	0.96	0.95	0.93
6	≥250, <280	26.0	1.00	0.99	0.99	0.99	0.98
7	≥280, <310	32.4	0.99	0.99	0.98	0.97	0.96
8	≥310, <340	38.1	1.00	1.00	1.00	0.99	0.99
9	≥340, <370	42.7	1.00	1.00	0.99	0.99	0.98
10+	≥370	100.0	1.00	1.00	1.00	0.99	0.99

* *E* = 0.13, the image-level error rate of the random forest classifier, tested with the test set.

## Data Availability

Data sharing is not applicable to this article.
